# Integrating ethnobiological knowledge into biodiversity conservation in the Eastern Himalayas

**DOI:** 10.1186/s13002-017-0148-9

**Published:** 2017-03-29

**Authors:** Alexander R. O’Neill, Hemant K. Badola, Pitamber P. Dhyani, Santosh K. Rana

**Affiliations:** 1 Fulbright-Nehru Research Scholar, United States-India Education Foundation (USIEF) and the United States Fulbright Commission, Washington, DC, USA; 2 G. B. Pant National Institute of Himalayan Environment and Sustainable Development, Sikkim Unit, Pangthang, Gangtok, East Sikkim, Sikkim 737 102 India; 3 G. B. Pant National Institute of Himalayan Environment and Sustainable Development, Kosi-Katarmal, Almora, Uttarakhand 263 643 India; 4 Central Department of Botany, Plant Systematics and Biodiversity, Tribhuvan University, Kirtipur, Kathmandu 44618 Nepal

**Keywords:** Biocultural diversity, Ethnobotany, Local ecological knowledge, Traditional knowledge, Sikkim, India

## Abstract

Biocultural knowledge provides valuable insight into ecological processes, and can guide conservation practitioners in local contexts. In many regions, however, such knowledge is underutilized due to its often-fragmented record in disparate sources. In this article, we review and apply ethnobiological knowledge to biodiversity conservation in the Eastern Himalayas. Using Sikkim, India as a case study, we: (i) traced the history and trends of ethnobiological documentation; (ii) identified priority species and habitat types; and, (iii) analyzed within and among community differences pertaining to species use and management. Our results revealed that Sikkim is a biocultural hotspot, where six ethnic communities and 1128 species engage in biocultural relationships. Since the mid-1800s, the number of ethnobiological publications from Sikkim has exponentially increased; however, our results also indicate that much of this knowledge is both unwritten and partitioned within an aging, gendered, and caste or ethnic group-specific stratum of society. Reviewed species were primarily wild or wild cultivated, native to subtropical and temperate forests, and pend IUCN Red List of Threatened Species assessment. Our results demonstrate the value of engaging local knowledge holders as active participants in conservation, and suggest the need for further ethnobiological research in the Eastern Himalayas. Our interdisciplinary approach, which included rank indices and geospatial modelling, can help integrate diverse datasets into evidence-based policy.

**Electronic supplementary material:**

The online version of this article (doi:10.1186/s13002-017-0148-9) contains supplementary material, which is available to authorized users.

## Background

Conservation practitioners have historically considered the role of human communities only or primarily in terms of the threats that extractive and transformative activities pose on the environment [1–3]. As a theoretical consequence, people-free or ‘fortress conservation’ strategies have become the dominant means of protecting ‘natural’ systems from anthropogenic influence [4, 5]. However, over the past two decades, a paradigm shift among conservationists has challenged this convention [6–8]. Termed *biocultural approaches to conservation* [9], recent programs have integrated the innovations, practices, and worldviews of Indigenous and local communities into policies addressing the rapid attrition of Earth’s biological and cultural diversity, hereafter termed *biocultural diversity* [9–12].

Thematically, biocultural approaches to conservation emphasize the dynamic, multi-scalar feedback loops that link social and ecological processes [9]. They synthesize biodiversity science and ethnographic fieldwork to discern processes that shape extant Earth systems [13]. In doing so, they help deconstruct the dualism separating ‘nature’ from society, and place local people back in parks as conservation agents [14–16]. Worldwide, such projects have had a variety of reported successes, including heightened spiritual connection and increased environmental literacy [16, 17]. However, debates continue as to the verity of reported claims and the extent to which conservation programs should serve human welfare [9].

‘Landscape’ initiatives in the Eastern Himalayas evidence the successful utilization of biocultural principles for conservation purposes [18]. In 1997, an International Centre for Integrated Mountain Development (ICIMOD)-led collective petitioned for the designation of Mt. Khangchendzonga as a dynamic complex of socio-ecological interaction [19, 20]—a *biocultural hotspot*. The transboundary Khangchendzonga Landscape (KL) is situated within the Himalayan Biodiversity Hotspot [21, 22], and includes Bhutan, India, and Nepal. It incorporates 7.2-million people belonging to diverse ethnic communities, including Indigenous groups like the Lepchas of Sikkim and Darjeeling, the Lhop (Doya) of Amu Mo Chhu Valley, and Walungpas of Walangchung Gola of Taplejung [22]. Because of this complexity, biocultural approaches to conservation facilitated environmental management in the KL. Co-management, community-based conservation, and integrated conservation and development, for example, have empowered Indigenous and local peoples through non-government organizations (NGOs), and promoted international cooperation along sensitive geopolitical boundaries [21–24].

Since its original delineation, the India-led Khangchendzonga Landscape Conservation and Development Initiative and Feasibility Assessment has committed 14,061 km^2^ of land, with a population of 6,325,457 people, into KL conservation policies [25]. KL-India’s network is comprised of 16 protected areas (PAs), including a biosphere reserve (*n* = 1), national parks (*n* = 4), and national wildlife sanctuaries (*n* = 11). Within the Indian landscape, the Government of Sikkim’s (GoS) efforts in the Khangchendzonga Biosphere Reserve (KBR) are perhaps the greatest testament to claims regarding efficacy of biocultural principles for achieving local and international conservation objectives. Sikkim occupies a 7096-km^2^ zone of the Indian Eastern Himalayas, and has 37% of its total area, excluding transition zones of the KBR, designated for conservation purposes. The Khangchendzonga National Park (KNP) encompasses over 80% of all protected lands in Sikkim (Table 1); six additional sanctuaries can be found within the borders of these PAs [25]. For maintaining tribal sanctity and for cultural conservation purposes, the GoS demarcated Dzongu Territory for the exclusive use of the Indigenous Lepcha people [26]. On 17 July 2016, the KNP was inscribed India’s first mixed-criteria UNESCO World Heritage Site based on the region’s biocultural heritage.

**Table 1:** Protected areas (PAs) in the Sikkim Eastern Himalayas, and the potential number of species with ethnobiological records found in each based on reviewed altitudinal range data

Map ID	Protected Area	Year Established	District(s) Covered	Area (km^2^)	IUCN Category	Altitudinal Range (m)	Estimated Species with Ethnobiological Records
1	Khangchendzonga National Park	2007	North, West	1784	IV	1400–8598	920
2	Shingba Rhododendron Sanctuary	1992	North	43	IV	3048–4575	280
3	Maenam Wildlife Sanctuary	1987	South	35.34	IV	2000–3263	609
4	Fambonglho Wildlife Sanctuary	1984	East	51.76	IV	1524–2749	848
5	Kyongnosla Alpine Sanctuary	1992	East	31	IV	3292–4116	223
6	Barsey Rhododendron Sanctuary	1996	West	104	IV	2110–4100	560
7	Kitam Bird Sanctuary	2005	East	6	-	320–875	635
8	Pangolakha Wildlife Sanctuary	2000	East	128	IV	1760–4390	759

Reference Fig. 3 for geographical location of detailed PAs

Traditional and community knowledge buttresses conservation policies in Sikkim and is heralded for its adaptive capacity. However, at the same time, Sikkim’s biocultural heritage is threatened by ‘modernizing’ forces associated with globalization and rapid climatic change. As noted in the Sikkim Biodiversity Action Plan, the state lacks formalized and collated records of its biodiversity, which extends into ethnobiological documentation [27]. Even among existing studies, including ethnobiological datasets, records are strictly qualitative, and exist as repetitive, fragmentary notes that lack a consolidated attempt for strengthening policy [28, 29]. Mobilizing this knowledge and associated datasets into environmental management programs remains a challenge.

In this review, we explore the application of ethnobiological knowledge for biodiversity conservation in Sikkim. Specifically, we ask: (i) What is the spatio-temporal pattern of ethnobiological knowledge documentation?; (ii) How is ethnobiological knowledge partitioned within and among ethnic communities?; (iii) What species are priority targets for conservation, and are these species found within protected areas? In asking these questions, we hope to reframe discourses that focus on the Eastern Himalayas as only or primarily a reservoir of biological and genetic diversity. To our knowledge, our manuscript serves as the first ethnobiological review of the Sikkim Eastern Himalayas.

## Methods

### The Sikkim Eastern Himalayas

Sikkim is divided into four districts, and situated between Bhutan, Nepal, the Tibetan Autonomous Region of China (TARC), and the Indian State of West Bengal. In the 17th Century, Lepcha and Bhutia communities established Sikkim as a Buddhist monarchy under kings termed *Chogyals. Chogyals* ruled for approximately 350 years until multi-directional process of change resulted in Sikkim’s protectorate status and eventual integration into India as its 22nd state in 1975 [30]. Prior to integration, major socio-ecological changes followed contact with the British East India Company in the mid-1880s. During this period, Nepali migration, here a generic term that includes many castes and ethnicities, was incentivized to promote colonial agricultural development in the Eastern Himalayas [31]. These progressive changes resulted in a rich admixture of ethnobiological traditions from the Greater Himalayas.

Demographic records from Sikkim have varied in quality since the first census in 1891; notwithstanding, Sikkim’s population appears to have increased from 30,458 to 607,688 people between 1891 and 2011 [32, 33]. The Anthropological Survey of India identified 25 ethnic communities in the state during its first ethnographic survey between 1988 and 1990 [34]. These communities are generally grouped as: (i) Bhutias (Lhopos, including Denjongpas, Lachenpas, and Lachungpas) and Lepchas, the autochthons of Sikkim who represent less than 20% of the total population; (ii) People of Nepalese origin, mainly Limbus and Rais, who began migrating to Sikkim from the 1870s and represent more than 75% of the population; and, (iii) People from the plains of India, including Bengalis, Biharis, and Marwaris [35]. In June 1978, Lepcha, Bhutia, Chumbipa, Dopthapa, Drokpa, Kagate, Sherpa, Tibetan, Tromopa, and Yolmo communities were recognized as Scheduled Tribes in Sikkim; the Kami, Damai, Lobar, Majhi and Sarki were classified as Scheduled Castes. The Government of India considers some ‘Nepali’ identifying or identified groups in Sikkim as ‘backward castes’: Gurung, Magar, Newars, Limbu/Subba, Rai, Sunwar, and Tamang. Bengali, Bihari, Deswali, Marwari, and Punjabi -identifying communities, all recent migrants who are diverse both within and among respective communities, are well-established in modern Sikkim [36]. In total, our study recognizes 32 ethnic communities, 17 languages, and 9 religions in Sikkim [34, 36–38].

Sikkim’s landscape is a well-recognized biodiversity hotspot, with habitat types broadly categorized into six categories that are correlated elevation (Table 2) [21]. However, extreme topographic variations and Sikkim’s horseshoe-shaped geography complicate these generalizations [27]. Some Global 200 Ecoregions found in Sikkim include Himalayan Alpine Meadows and Eastern Himalayan Broadleaf and Coniferous Forests [39]. Diverse assemblages of human communities living within and (re)producing these ecosystems have facilitated the region’s rich, biocultural heritage.

**Table 2:** Habitat zones in the Sikkim Eastern Himalayas, and some characteristic woody taxa with ethnobotanical records found within associated habitat zones (Adapted from [21, 53])

Habitat Zone	Forest Type		Some characteristic taxa with ethnobotanical records
Tropical (<1000 m)	(i)	Tropical riverine evergreen/deciduous Forest	*Bombax ceiba*, *Cycas pectinata*, *Dalbergia sissoo*,
	(ii)	Tropical Moist Evergreen/Deciduous Forest	*Dillenia indica*, *Duabanga grandiflora*, *Garuga pinnata*,
	(iii)	Tropical Moist Mixed Forest	*Lagerstromia speciosa*, *Mimosa pudica*, *Shorea robusta*
	(iv)	Tropical Dry Evergreen/Deciduous Forest	
Subtropical (1000–2000 m)	(i)	Subtropical Riverine evergreen/Deciduous Forest	*Callicarpa arborea*, *Castanopsis tribuloides*, *Fraxinus floribunda*,
	(ii)	Subtropical Moist Evergreen/Deciduous Forest	*Macaranga pustulata*, *Mangifera sylvatica*, *Pandanus furcatus*,
	(iii)	Subtropical Moist Mixed Forest	*Saurauia nepaulensis*, *Schima wallichi*
	(iv)	Subtropical Dry Evergreen/Deciduous Forest	
Warm Temperate (2000–2500 m)	(i)	Warm Temperate Riverine Evergreen/Deciduous Forest	*Alnus nepalensis*, *Castanopsis tribuloides*, *Engelhardia spicata*,
	(ii)	Warm Temperate Moist Evergreen/Deciduous Forest	*Evodia fraxinifolia, Ilex dipyrena*, *Juglans regia*,
	(iii)	Warm Temperate Moist Mixed Forest	*Lithocarpus pachyphyllus, Quercus lamellosa, Zanthoxylum acanthopodium*
	(iv)	Warm Temperate Dry Evergreen/Deciduous Forest	
Cool Temperate (2500–3000 m)	(i)	Cool Temperate Riverine Deciduous Forest	*Acer caudatum*, *Betula utilis*, *Cinnamomum impressinervium*
	(ii)	Cool Temperate Moist Evergreen Forest	*Cryptomeria japonica*, *Magnolia lanuginosa*, *Mahonia sikkimensis*,
	(iii)	Cool Temperate Moist Mixed Forest	*Rhododenron arboreum*, *Quercus lineata*
	(iv)	Cool Temperate Dry Evergreen Forest	
Subalpine (3000–4000 m)	(i)	Subalpine Riverine Evergreen Forest	*Abies densa, Abies spectabilis, Berberis insignis, Juniperus recurva,*
	(ii)	Subalpine Moist Evergreen Forest	*Larix griffithiana*, *Rhododenron barbatum*, *Rhododendron campanulatum, Taxus wallichiana*
	(iii)	Subalpine Moist Deciduous Forest	
	(iv)	Subalpine Dry Evergreen Forest	
Alpine (>4000 m)	(i)	Alpine Riverine	*Juniperus indica*, *Rhododendron fulgens*, *Rhododendron nivium*
	(ii)	Alpine Meadow	
	(iii)	Alpine Scrub	

### Data collection and standardization

From October 2015 through February 2016, we conducted a systematic review of publically available and accessible literature pertaining to ethnobiological knowledge in the Sikkim Eastern Himalayas. For this study, we defined ethnobiological knowledge as traditional and community knowledge—Indigenous and non-Indigenous—related to socio-ecological interactions between identified or identifiable taxa and the people of Sikkim. Using search terms Darjeeling/Kalimpong/Sikkim/Eastern Himalaya AND Ethno/Indigenous/Traditional, we searched four digital databases: (i) ENVIS [40]; (ii) Google Scholar; (iii) NELUMBO [41]; and, (iv) Project Muse [42]. We included Darjeeling and Kalimpong (West Bengal, India) as place-based keywords due to their historical association with the Kingdom of Sikkim. After analysis, we omitted data published in the ENVIS *Medicinal Plants of Sikkim* database due to its primary reference of non-Sikkimese user groups and medical traditions. We then conducted archival research at six institutions in Gangtok, Sikkim using the same criteria: (i) The Botanical Survey of India; (ii) The G. B. Pant National Institute of Himalayan Environment and Sustainable Development, Sikkim Unit; (iii) The Namgyal Institute of Tibetology; (iv) Home Department, Government of Sikkim Central Library; (v) Sikkim University Central Library; and, (vii) Sikkim State Bioinformatics Institute. Once collected, each source was reviewed for the following subsets of data: study site name, including the names of sacred landscapes, cities, villages, *panchyats*, *samitis*, blocks, districts, and subdivisions; bio-physical characteristics of site-specific studies; publication date; Indigenous and local castes, clans, and groups surveyed; and, species diversity. These sources are provided as an additional file [see Additional file 1].

We transcribed species data from each reviewed record into a working database [see Additional file 2]. After all sources were reviewed, we then standardized species to current taxonomic designations using international databases and field guides [43–48]. Concurrently, we tabulated the relative citation frequency for each species, and partitioned uses into one of 19 accepted categories (Table 2) [49, 50]. Relative citation frequency was calculated by dividing each citation value by the value of the most frequently cited species [see Additional file 2]. Regarding ecological data, we detailed Sikkim-specific altitudinal range data when possible [51–58]; data from the region were used as a proxy in the absence of Sikkim-specific records [45–47, 59–61]. Finally, we collected the following data: the conservation status of species from the IUCN Red List of Threatened Species [62] and Government of Sikkim [63]; and, naturalization, cultivation, or domestication status [47, 64]. Our study assumes that the number of use categories reported for a given species corresponds with the amount of attention it receives from communities in Sikkim. It is important to note that the number of uses might not correspond to current and active applications of those uses.

### Data analysis

We geo-referenced reported study sites and conducted spatio-temporal analyses of reviewed data in ArcGIS [11, 65, 66]. Specifically, we performed the following assessments: (i) identification of administrative districts with the highest representation of ethnobiological records; (ii) temporal analysis of ethnobiological knowledge documentation; and, (iii) identification of surveyed communities and their knowledge documentation through time.

We calculated two conservation ranks for reviewed species based on accepted methods for categorical data (Tables 3 & 4): Harvest Rank (HR) and Sensitivity Rank (SR) (see [67, 68]). From this point, however, we could not carry out further statistics as our rank assignation was based on qualitative criteria in which numerical ranks represent other categories rather than quantities. The HR value incorporated harvest and provenance data (Table 4): wild and native (WN) = 5; wild-cultivated and native (WCN) = 4; wild and non-native (WNN) = 3; wild-cultivated and non-native (CNN) = 2; and cultivated (C) = 1 [60, 67, 69]. We believe that wild and native species are of higher conservation priority because on their provenance in the Sikkim Eastern Himalaya, and their historical role in regional ecology. The SR value accounted for three important factors determining the conservation status of species: mode and extent of harvesting; altitudinal range, or amplitude; and, (iii) species’ population status, based on IUCN Red List of Threatened Species and Government of Sikkim recommendations [60, 63]. Using matrix criteria to account for these attributes, we scored SR in a decreasing order to 8-1 (Table 4) [69]. We also calculated a relative citation frequency (CF), or the number of reviewed citations for species *e* divided by the maximum number of citations for *n* surveyed species. These data are provided as an additional file [see Additional file 2]. We hope these ranks, although qualitative, serve as platform for future analyses that integrate social and natural science data with community knowledge to indicate priority targets for biodiversity conservation.

**Table 3:** Criteria for ranking species for Sensitivity Rank (SR) of reviewed species

Attribute	Attribute Criteria
Species Engagement	
High Relative Intensity (D)	Harvesting/utilizing either (i) whole animal or animal part in a manner that reduces animal’s lifespan (*i.e.* bones, ivory, meat, *etc.*); or (ii) whole plant, rootstock, rhizome, fungal body, *etc.*
Low Relative Intensity (N)	Species engagement excluding the above
Altitudinal Range	
Restricted (R)	Range limited to one habitat zone
Wide (W)	Range extending to two or more habitat zones (Refer to Table 2 for habitat zones)
Population Status	
Threatened (T)	IUCN or Government of Sikkim-recommended Critically Endangered (CR), Endangered (EN), or Vulnerable (VU)
Not Threatened (U)	IUCN or Government of Sikkim-recommended Near Threatened (NT), Least Concern (LC), or Not Assessed (NA)

**Table 4:** The structure of our sensitivity matrix used to rank reviewed species [60, 67–69]

Sensitivity Rank (SR)	Extraction	Occurrence	Population status
8	D	R	T
7	D	R	U
6	D	W	T
5	D	W	U
4	N	R	T
3	N	R	U
2	N	W	T
1	N	W	U

*Abbreviations*: (i) Extraction: Destructive Harvesting (D) or Non-destructive Harvesting (N); (ii) Occurrence: Rare (R) or Widespread (W); (iii) Population Status: Threatened (T) or Unthreatened (U). Refer to Table 3 for further elaboration

Using the altitudinal range of each reviewed species, we modeled biocultural hotspots in Sikkim using a standard methodology at 100 m altitudinal resolution (Fig. 2) (see [70]). Here, we aimed to project a qualitative map that identified priority regions for biodiversity conservation based on the altitudinal range of reviewed species. We acknowledge that modeling procedures often account for GIS-based, site-specific occurrences and bioclimatic variables associated with specific species [70, 71]. However, such data from Sikkim is only available in heterogeneous, fragmented forms that are geographically biased or incorrect. Moreover, Sikkim’s topography, which averages 40° slope, and altitudinal variation, ranging from 284 m to 8586 m, generate a plethora of unpredictable microhabitat and microclimatic conditions that: influence species distributions; limit the practicality of field surveys; and, bias conventional modeling procedures [72]. Therefore, in the absence of data, our model engages altitudinal distribution data as the sole proxy for various methodologies [73].

## Results

### Spatio-temporal analysis

**Fig. 1:**
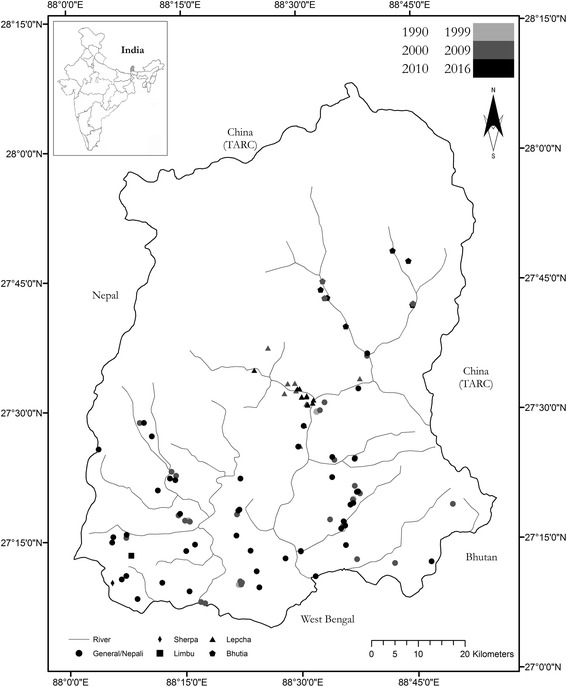
Spatio-temporal review of site-specific records in the Sikkim Eastern Himalayas

Our review resulted in 176 ethnobiological records from the Sikkim Eastern Himalayas [see Additional file 1]. These records include 42 site-specific surveys, 18 of which were multi-site studies (total geo-referenced locations: 119), 94 contained methodological or instructional content on species use, 15 contained folk tales or cultural information beyond medical or material utility, and ten were biodiversity-related records with ethnobiological footnotes (Fig. 1). Based on site-specific records, North District received the greatest survey effort (37%) followed by West District (33%), East District (16%), and South District (14%). North District’s survey effort was driven by studies in Dzongu Territory (North District), a once-royal land plot now reserved for certain Lepcha families. The average survey altitude across site-specific records was 1775 m ASL (+/- 712 m SD).

**Fig. 2:**
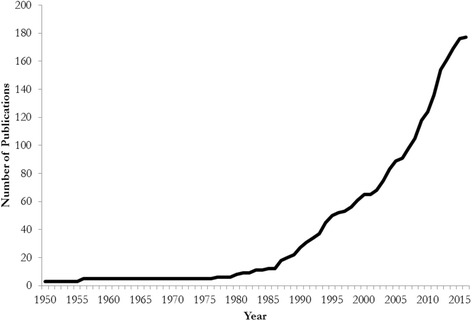
Publication of ethnobiological records from 1950 to 2016

Our temporal analysis revealed that biocultural records were first published during the East India Company’s expansion across the Indian Subcontinent during the 1840s [74–76]. Our review specifies that Sir Archibald Campbell, the first British political officer to Sikkim and Darjeeling, authored the first ethnobotanical vignettes from Sikkim as they relate to Lepcha communities [74]. Concurrently, Sir Joseph Dalton Hooker, a British doctor and an esteemed naturalist, reflected upon the region’s biocultural heritage in his two-volume *Himalayan Journals* published 1854. For much of the mid-19th Century, naturalist records were the primary sources of biocultural anecdotes, particularly as they relate to *Rhododendron* spp. (see [21]). We identified no biocultural records dating one century after Hooker’s *Journals*. A few generalist surveys were conducted during the mid-20th Century in what was termed the Sikkim and Darjeeling Hills [77, 78]. Approximately 14% of all ethnobiological records from Sikkim were published between 1854 and 1990, none of which detailed specific study sites (Fig. 2). The late 20th Century saw an exponential increase in these studies across Sikkim, particularly regarding medicinal plant species. Place and district-specific studies revealed that the earliest documentation began in West District, and moved toward North District during the second decade of the 21st Century (Fig. 1). Approximately, 87% of all reviewed biocultural studies were published between 1990 and 2016, with a significant increase in publication rate during the first decade of the 21st Century (*r* = 0.863; *P* < 0.001).

### Surveyed ethnic communities

Six of 32 identified ethnic communities in Sikkim have written ethnobiological records: Bhutia, Lepcha, Limbu, Nepali, Sherpa, and Tibetan communities. However, most reviewed records were not ethnic-group specific and reported data and/or knowledge in Sikkim-vernacular Nepali language. Nepali-identified or identifying groups, including non-specific reports detailed in Nepali language, had the greatest number of identified species (732), followed by Lepchas (377), Limbus (298), Tibetans (120), Bhutias (74), and Sherpas (35). Four categories of male specialized users and spiritual healers were reported from four ethnic communities: Bhutia: *Lama*; Lepcha: *Bomthing*, *Mon-bomthing*; Nepali: *Bijuwa*, *Fedangwa*, *Jhakri*; and, Tibetan: *Amchis*.

### Species diversity

A total of 1128 species distributed across three kingdoms, 213 families, and 712 genera have ethnobiological records in the Sikkim Eastern Himalaya [see Additional file 2]. Plantae was the most surveyed kingdom (995 species; 625 genera; 160 families), housing 88% of all reviewed species. Animalia (species: 76; genera: 50; families: 28; 7% of reviewed species) and Fungae (species: 57; genera: 37; families: 25; 5% of reviewed species) exhibited a comparative dearth of records [see Additional file 2]. Across kingdoms, 105 species were considered Himalayan or Eastern Himalayan endemic [45, 79]. The average range amplitude of species in Animalia was 1098 m (+/- 1009 m SD), in Fungae 1683 m (+/- 884 m SD), and in Plantae 1329 m (+/- 584 m SD).

In Plantae, angiosperms were the most explored discipline, accounting for 96% of all documented plant species (957). Pteridophytes (23), Gymnosperms (10), and Bryophytes (5) received notably less attention in comparison [27]. Five plants were reported as endemic to the Sikkim Eastern Himalayas, namely: *Aconitum ferox* var. *naviculare*, *Allium sikkimense*, *Mahonia sikkimensis*, *Rhododendron sikkimensis*, and *Swertia pedicellata*. The taxonomic designations for these and many of our reported species, however, are pending official revision. In Animalia, fish were the most represented Chordates (37), followed by Mammals (22), Birds (12) and Amphibians (3) [see Additional file 2]. Three invertebrates were cited, two of which were in the Apidae. In Fungae, Basidiomycetes accounted for 77% of reviewed species (44), with approximately 85% of the remaining 13 Ascomycetes being Lichens.

### Ethnobiological uses

Across kingdoms, species were primarily used as medicine to prevent or manage gastro-intestinal afflictions, dermatological conditions, and respiratory-tract infections (Table 5) [see Additional file 2]. The ten most cited species also occupied the most diverse use categories, respectively: *Swertia chirayita* (Gentianaceae), *Bergenia ciliata* (Saxifragaceae), *Oroxylum indicum* (Bigoniaceae), *Uritica dioica* (Urticaceae), *Acorcus calamus* (Acoraceae), *Nardostachys jatamansi* (Caprifoliaceae), *Rhododendron arboretum* (Ericaceae), *Rumex nepalensis* (Polygonaceae), *Astilbe rivularis* (Saxifragaceae), and *Cheilocostus speciosus* (Costaceae). Species-wise data are available as an additional file [see Additional file 2].

**Table 5:** The distribution of reviewed species across 19 ethnobiological categories

Ethnobiological	Category	Category elaboration		Linnaean Kingdom		
Category	Abbreviation	Affliction(s)/Disorder(s)/Use(s)	Total	Animalia	Fungae	Plantae
Antidote	ANTI	Treatment for Animal Venom, Fish Stupefying	70	2	0	68
Behavioral and Mental Health	MENT	Hysteria, Mental and Nervous Disorders	24	0	0	24
Circulatory Health	CIRC	Bleeding, Blood Health, Hemorrhage, Lymphatic System	133	2	1	130
Antiseptic, Dermatological Health	DERM	Abrasions, Burns, Boils, Skin Diseases and Parasites, Hair Problems	373	11	0	362
Dental and Oral Health	ORAL	Throat Infection, Toothache, Oral Infection	99	2	0	97
Ear and Mastoid Health	HEAR	Earache, Hearing Deficiency, Vertigo	21	0	0	21
Endocrine and Metabolic Health	EDCR	Diabetes, Hormonal Disorder	144	3	2	139
Gastro-intestinal Health	DGST	Diarrhea/Dysentery, Indigestion, Laxative, Nausea, Parasites, Vomiting	409	17	1	391
Genito-urinary Health	URIN	Bladder and Renal Infections, Sexually Transmitted Infections, Urinary-tract Infections	181	0	1	180
Hepatic Health	HEPT	Jaundice, Liver Disorders	106	1	2	103
Musculo-skeletal Health	SKEL	Antispasmodic, Body Aches, Sprain, Fracture, Rheumatism/Arthritis	105	2	0	103
Neoplasm Treatment	NEOP	Cancer, Growths, Moles, Tumors	16	0	0	16
Nervous System Health	NERV	Epilepsy, Memory, Migraine, Stimulant	97	0	1	96
Ophthalmic Health	OPTH	Adnexa, Eye Disease	48	0	0	48
Pregnancy, Childbirth, Perinatal Care	PREG	Delivery, Lactation, Menstruation, Pregnancy	52	1	0	51
Respiratory Health	RESP	Allergy, Asthma, Bronchitis, Pneumonia,	206	11	9	186
Alimentary Purpose(s)	FOOD	Edible, Food, Butter, Oil, Flour, Pickle, Dry Fruit, Candies	452	50	48	354
Cultural or Spiritual Use	CULT	Culture-specific Disease, Folktales, Legends, Ritual Ingredients, Spirituality	35	9	0	26
Material	MTRL	Art, Construction, Dye, Fodder, Handicrafts, Instrument, Utensils	169	7	0	162
Veterinary Medicine	VETN	Ethno-veterinary Medicine	105	0	0	105

Disease and health-related categories were based on criteria outlined by the World Health Organization [61]

### Conservation ranks

Wild (CR 5 and 3; 922 species; 82% total) and native taxa (CR 5; 817; 72%) were cited more frequently than wild-cultivated (CR 4 and 2; 107; 10%) and cultivated species (CR 1; 99; 8%). Most species were harvested, cultivated, or used in a sustainable manner (SR 1-4; 642; 57%) and exhibited altitudinal distributions that crossed multiple habitat types (SR 6-5, 21; 962; 85%). Of 1128 species, approximately 80 species were both destructively harvested and had restricted ranges. Four of these species also had a threatened status in Sikkim (SR 8), including *Cymbidium grandiflorum* (Orchidaceae), *Flickingeria fimbriata* (Orchidaceae), *Ophiocordyceps sinensis* (Ophiocordycipitaceae), and *Tor putitora* (Cyprinidae). Based on IUCN Red List of Threatened Species assessments [60], three of all species were Critically Endangered (CR), four are Endangered (EN), seven are Vulnerable (0.64; VU), 11 are Near Threatened (1.00% NT), 99 are of Least Concern (9.03% LC), and 972 species have not been assessed (88.69% NA) [see Additional file 2]. An additional 25 species have recommended conservation statuses by the Government of Sikkim based on IUCN-CAMP criteria ([63]; see Additional file 3). These species include exploited medicinal plants such as *Swertia chirayita*, *Nardostachys jatamansi*, *Picrorhiza kurroa* (Plantaginaceae), *Sinopodophyllum hexandrum* (Berberidaceae), and *Valeriana jatamansi* (Caprifoliaceae).

### Biocultural hotspots

**Fig. 3:**
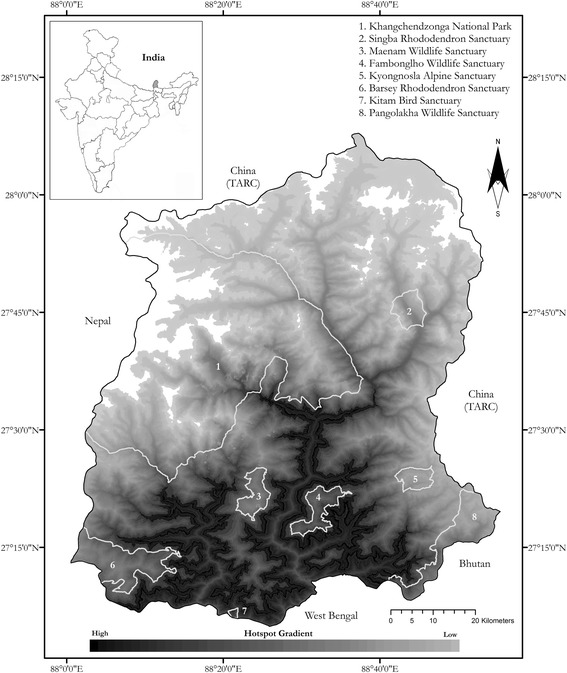
Biocultural hotspots in the Sikkim Eastern Himalayas

Our map illustrates areas that have the greatest potential richness of species with biocultural records, termed biocultural hotspots, based on the elevational range of species (Fig. 3). Grid cell values range 7 (low) to 619 (high) species and are presented at 100-m elevational resolution. The highest grid-cell values were located outside of PAs. Richness of culturally important species was highest in subtropical zones across kingdoms, with a sharp decline toward alpine regions.

## Discussion

Our review indicates that Sikkim has a rich biocultural heritage that includes knowledge pertaining to over 1100 species of animals, fungi, and plants. Local people not only know about the useful properties of these species, but also the community ecology and life histories of diverse organisms [80]. These aspects of ethnobiological knowledge, which encompass abundance, distribution, and phenology, significantly influence community management practices and can therefore benefit conservation planning in Sikkim [29, 79]. For instance, in previous studies, Lepcha communities were engaged by government researchers to understand the population status of under-surveyed bird species; local communities were found to provide “data” at the accuracy needed to make management decisions [81, 82]. Our reviewed records also implied that faith traditions and community taboos sustain many ethnobiological relationships in Sikkim, and cultivate a sense of stewardship toward critical habitat [35, 83–86]. We conclude that targeting biocultural knowledge systems, including gaps in ethnobiological research, is a practical way to incorporate local peoples—their knowledge, land, and participation—into multi-scalar conservation directives in the Eastern Himalayas.

Our analyses illuminated the dynamic nature of ethnobiological knowledge, and evidenced its ongoing construction amidst changing socio-ecological conditions. We traced a dramatic increase in ethnobiological records published since the 1950s, with a significant spike in the mid-1990s. This trend appears correlated with both the relaxation of permit restrictions into Sikkim, and the 1993 initiation of the Convention on Biological Diversity (CBD) following the Earth Summit in Rio de Janeiro in 1992. The CBD obliged signatory polities, including India, to acknowledge and preserve biocultural knowledge as an adaptive resource for conservation initiatives, record and disseminate biocultural knowledge for practical applications, and ensure equitable benefits arising from biocultural knowledge (reviewed by [21]). Our assessment highlighted, however, a stark difference in ethnobiological knowledge within and among ethnic communities. Intra-cultural differences compounded overall knowledge diversity based on age, gender, occupation, and individual strategies and interests [26]. These factors were not quantitatively justified in any of our reviewed studies. The paucity of records from 26 identified ethnic communities further indicates that much of Sikkim’s biocultural heritage remains as unwritten, oral traditions situated within a gendered, caste-specific, and aging stratum of society. In the future, we suggest that researchers begin addressing these gaps through collaborations with nomadic or semi-nomadic peoples at high altitudes, including Bhutia, Chumbipa, Dopthapa, Drokpa, Kagate, Sherpa, Tromopa and Yolmo communities. High-altitude zones are particularly sensitive to climatic changes that may alter community assemblages, ecological processes, and, as an extension, historical ways of relating to the environment [85, 87, 88]. With the participation of these groups, conservationists can craft more holistic and culturally appropriate strategies for both restoration and conservation in the Eastern Himalayas.

As previously alluded to, older members of rural communities were the primary user group of reviewed species, namely for medicine. Knowledge of species use, practice, and folklore was reported to decrease in recent generations, as much of the knowledge was documented from collaborators between 50 and 70 years of age [26, 29]. Today, younger generations migrate to urban centers where they are neither exposed to local species nor the traditions that surround them. Both imposed legal structures in the early 21st Century and market liberalization in the mid-1990s have further complicated knowledge transmission and species use, resulting in the attrition of ethnobiological knowledge in Sikkim. For instance, between the 1970s and 1990s, the Sikkim Forest, Environment and Wildlife Management Department permitted commercial exploitation of medicinal plants, including from PAs. However, as of 2001, the government implemented a 5-year ban on medicinal plant collection via Order No. 13/F/Env&W. This order received a 5-year extension in 2006, and will likely be ratified again in the future. Moreover, local access to state healthcare providers and allopathic medicine has reduced local dependency on wild animals, fungi, and plants as medicine.

Despite the decreased reported use of medicinal plants, many wild species remain a vital part of Sikkimese cuisine [89–94]. Local communities have regular access to wild edibles at markets in the cities of Gangtok (East District), Geyzing (West District), Namchi (South District), and Singtam [91, 93, 95]. Various ferns (*Diplazium* spp.; Athyriaceae), the Sikkim Cobra Lily (*Arisaema utile*; Araceae), and Stinging Nettles (*Uritica dioica*; Urticaceae) were some frequently cited edibles, and were often prepared alongside pickled vegetables, like Nodding Tupistra (*Tupistra nutans*; Asparagaceae), in traditional Sikkimese cuisine [96–99]. Fruits from Bastard Oleaster (*Elaeagnus latifolia*; Elaeagnaceae), Burmese Grape (*Baccaurea ramiflora*; Phyllanthaceae), and *Machilus edulis* (Lauraceae) were also seasonal favorites with high reported consumption [100–102]. Regarding Fungae, commercial cultivation has increased in lower-altitude zones, particularly of species in the genera *Agaricus* and *Pleurotus* [51, 52, 103]. Wild animals were also consumed for medicinal purposes, albeit at low reported frequency and only in rural contexts. The meat of Asian Black Bear (*Ursus thibetanus*; Ursidae), Bengal Fox (*Vulpes bengalensis*; Canidae), Central Himalayan Langur (*Semnopithecus schistaceus*; Cercopithecidae), Himalayan Crestless Porcupine (*Hystrix brachyura*; Hystricidae), and Hodgson’s Giant Flying Squirrel (*Petaurista magnificus*; Sciuridae) were reported to treat respiratory diseases, namely Tuberculosis, which are common in Sikkim [87–104]. Fish are available in most market places, and have high reported consumption; however, their populations likely face threats from hydroelectric dam development in Sikkim [105, 106]. Future studies should quantify the value and quantity of wild edibles sold in marketplaces to better understand anthropogenic pressures on wild populations and expand wild-cultivation practices when possible.

Incentivizing and/or commercializing the cultivation of edible and medicinal plants and fungi within agroforestry systems may reduce pressure on wild populations and create habitat corridors for threatened species [29, 107]. To date, most conservation efforts in Sikkim have been directed toward the nominal designation of PAs at mid and high altitudes. Military encampments and agricultural landscapes surround these PAs. Moreover, traditional doctors, spiritual healers, and rural villagers still harvest and collect many reviewed species from these areas despite legal restrictions [104, 108–112]. Conservationists can begin addressing these pressures, without marginalizing local communities, by promoting cooperative agroforestry programs along PA borders. Recent reviews, for example, have suggested integrating edible and medicinal plant cultivation into existing Nepalese Paperbush (*Edgeworthia gardneri*; Thymelaeaceae) or Black Cardamom (*Amomum subulatum*; Zingiberaceae) agroforestry systems which already yield high profits [29, 113, 114]. As noted by Charnley et al. [115], such programs must do more than identify the “right” or best model for knowledge application and sharing, and must address existing societal factors that may hinder program implementation or undermine community structures. Organizational frameworks could, therefore, draw upon pre-existing *dzumsa* and *dwichi* committee structures in Sikkim, which have legacies of conservation impact [116, 117]. Any cooperative, however, must ensure that agroforestry systems produce marketable amounts of edibles that can either be preserved or transported to market before spoilage (as reviewed by [29, 116]). Our conservation rank system and additional files is useful for identifying target species based on criteria of interest, including medicinal use, altitudinal range, and population status [see Additional files 1, 2 and 3].

Incorporating ethnobiological knowledge into biodiversity conservation is a meaningful way to empower local communities to both monitor and preserve species of biocultural importance [9, 11, 116]. Based on our review, communities have obvious incentive to conserve biodiversity for cultural purposes and practical use. However, our results suggest a literature bias toward medicinal plants, and a paucity of records from the kingdoms Animalia and Fungae. To hone the applicability of our biocultural hotspot concept, we recommend that researchers incorporate new criteria, including species-specific ranges and habitat information, into our model structure. Moreover, we suggest that researchers document ethnobiological relationships that extend beyond medicinal uses of species to include living oral traditions, folklore, art, *etc*. By combining ethnobiological surveys with biodiversity science, particularly the gaps noted in recent reviews [21, 29], conservationists can better understand the socio-ecological dynamics shaping modern Sikkim.

## Conclusion

We collated and applied ethnobiological knowledge to promote biodiversity conservation in the Eastern Himalayas. We began with a spatio-temporal review of biocultural records from Sikkim, India to understand: (i) patterns in biocultural knowledge documentation; (ii) the diversity of species with biocultural records; and (iii) the partitioning of biocultural knowledge within and among ethnic communities. We then galvanized these records into two conservation indices and a biocultural hotspot model that indicate conservation priorities in Sikkim.

## Additional files

Additional file 1: Ethnobiological records reviewed by this manuscript. (PDF 147 kb)

Additional file 2: Reviewed species with ethnobiological records, including rank values, distributional data, ethnobiological uses. (XLSX 126 kb)

Additional file 3: Some threatened species in the Sikkim Eastern Himalaya. (XLSX 12 kb)
