# Supplementary material for: Integrating ethnobiological knowledge into biodiversity conservation in the Eastern Himalayas
Source: J Ethnobiol Ethnomed. 2017 Mar 29;13:21. doi: 10.1186/s13002-017-0148-9 (PMC5372287; doi:10.1186/s13002-017-0148-9)
Supplement: Supplementary file 1 — Ethnobiological records reviewed by this manuscript. (PDF 147 kb) [file 13002_2017_148_MOESM1_ESM.pdf]

Additional File I

Bibliography of reviewed biocultural records from Sikkim

1. Acharjee M, Roy D. 2013. Rong ichthyological knowledge: a leeway. King Gaeboo Aachyok. 2013;4:25-34.
2. Acharya BK, Chettri B, Vijayan L. Indigenous knowledge of Lepcha community for monitoring and conservation of birds. Ind J Tradit Knowl. 2009;8(1):65-9.
3. Agrawala DK, Purohit CS, Das K. A review of ethno-medicinal orchids in Sikkim Himalaya. In: Kumar S, editor. Ethnobotanical studies in India. New Delhi: Deep Publications; 2014. p. 201-10.
4. Agarwal S, Thapa R, Srivastava T. Some industrially important aromatic plants of Sikkim Himalayas. Ind Perf. 1987;31(2):113-5.
5. Ansari AA, Panda AK, Brahma KK. Medicinal uses of flowering plants by the local people of Samdong. JDRAS. 2009;30(3-4):41-8.
6. Avasthe RK, Kumar A, Rahman H. Edible horticultural crop diversity in Sikkim Himalaya. In: Sikkim biodiversity: significance and sustainability. Gangtok: Sikkim State Council of Science and Technology; 2012. p. 20-32.
7. Badola HK, Pradhan BK. Plants used in healthcare practices by Limboo tribe in south-west of Khangchendzonga Biosphere Reserve, Sikkim, India. Indian J Tradit Knowl. 2013;12(3):355-69.
8. Badola HK, Pradhan BK. Economic viability of cultivation of *Swertia chirayita*, a high value endangered medicinal herb in Himalaya. ZAG. 2007;16(3):118-24.
9. Badola HK, Pradhan BK, Subba S, Rai, LK, Rai YK. Sikkim Himalayan Screw-pine, *Pandanus nepalensis*: a much neglected and underexploited NTFP. On-wood News. 2009;18:44-5.
10. Bam J, Rai S, Bhattacharya D, Maiti S, Pathak P, Bera AK, et al. Indigenous curative and prophylactic traditional practices used against hematophagous leeches in Arunachal Pradesh and Sikkim. Indian J Tradit Knowl. 2015;14(3):493-7. URL: http://14.139.47.15/handle/123456789/32108.
11. Basnett H, Mohanty J, Shrestha B, Pal P. Ethnomedicinal plants used for the treatment of sprains and fractures by the Nepalese community of East Sikkim. Uni J Pharma Sci Res. 2015;1(1):10-4.
12. Basu K. Common medicinal plants of Darjeeling and Sikkim. Kolkata: West Bengal Press; 1956.
13. Basu P, Mitra B. A note on the lesser-known plant *Bischofia javanica* (Bischofiaceae) in Sikkim. J Econ Taxon Bot. 1999;15(3):703-4.
14. Bennet SSR. Ethnobotanical studies in Sikkim. Indian Forest. 1983;109(7):477-81.
15. Bennet SSR. Ethnobotanical studies in West Sikkim. J Econ Taxon Bot. 1985;7:317-21.
16. Bharati K. Folk veterinary medicinal plants of Sikkim Himalayas: ethnoveterinary knowledge of Sikkim Himalayas. Saarbrücken: Lambert Academic Publishing; 2011.
17. Bharati KA, Sharma BL. Studies on ethnoveterinary uses of plant resources of Sikkim. Indian Forest. 2009;135(5):691-6.
18. Bharati KA, Sharma BL. Some ethnoveterinary plant records for Sikkim Himalaya. Indian J Tradit Knowl. 2010;9(2):344-6.
URL: http://nopr.niscair.res.in/handle/123456789/8169.
19. Bharati KA, Sharma BL. Plants used as ethnoveterinary medicines in Sikkim Himalayas. Ethnobot Res Appl. 2012;10:339-56.
URL: http://libojs3.lib.sfu.ca:8114/era/index.php/era/article/viewArticle/606.
20. Bhasin V. Ecology, culture, and change: tribals of Sikkim Himalaya. New Delhi: Inter- India Publications; 1989.
21. Bhasin V. Medical anthropology: healing practices in contemporary Sikkim. In: Bhasin V, Bhasin M, editors. Anthropology today: trends, scope and applications. Vol. 3. New Delhi: Kamala-Raj Enterprises; 2007. p. 59-94.
22. Bhasin V. Pastoralists of Himalayas. J Biodivers. 2013;4(2):83-113.
23. Bhasin V, Srivastava VK. Ecology and culture of the Bhutias of North Sikkim. J Human Ecol. 1990;1(3):277-85.
24. Bhutani K. Herbal wealth of North-east India: database and appraisal. Punjab: Department of Natural Products, National Institute of Pharmaceutical Education and Research; 2008.

Additional File I
Bibliography of reviewed biocultural records from Sikkim

25. Biswas K. Common medicinal plants of Darjeeling and the Sikkim Himalayas. Alipore: West Bengal Government Press; 1956.
26. Bole P. Aromatic plants of Sikkim: a preliminary study in utilization. In: Himalaya, Ecologie, Ethnologie. Paris: C.N.R.S; 1977.
27. Borah TR, Avasthe R, Helim R. Large cardamom (*Amomum sublatum*): a traditional cash crop of Sikkim. Asian Ag Hist. 2012; 16(3): 271-7.
28. Borah TR, Helim R, Gogoi R, Kumar A. Versatile uses of maize in Sikkim. Asian Ag Hist. 2012;16(2):211-5.
29. Borah TR, Rahman H. Mushrooms in biodiversity and food security of Sikkim. In: Arrawatia ML, Tambe S, editors. Biodiversity of Sikkim: exploring and conserving a global hotspot. Gangtok: Department of Information and Public Relations, Government of Sikkim; 2011. p. 29-42.
30. Campbell A. Note on the Lepchas of Sikkim, with a vocabulary of their language. J Asian Soc Bengal. 1840;9:379-93.
31. Chanda R, Mohanty J, Bhuyan N, Kar P, Nath L. Medicinal plants used against gastrointestinal tract disorders by the traditional healers of Sikkim Himalayas. Indian J Tradit Knowl. 2007;6(4):606-10.
32. Chauhan A. Ethnobotanical studies in Sikkim Himalaya. In: Singh G, Singh H, Mukherjee, editors. Ethnomedicine of North-east India. Guwahati: National Institute of Science Communication and Information Resources; 2003. p. 199-214.
33. Chettri A, Barik SK. Assessing ethnobotanical value and threat status of Tetrastigma rumicispermum (Lawson) Planch, a lesser known liana species of Khangchendzonga Biosphere Reserve, Sikkim. Ind J Trad Know. 2013; URL: http://14.139.47.15/handle/123456789/16852.
34. Chettri R, Rai B, Khawas DB. Certain medicinal plants in the folklore and folklife of Darjeeling and Sikkim Hills, India used for the treatment of ailments in domestic animals. J Econ Taxon Bot. 1992;10:393-8.
35. Chettri N, Sharma E. A scientific assessment of traditional knowledge on firewood and fodder values in Sikkim, India. Forest Ecol Manage. 2009; doi:10.1016/j.foreco.2009.02.002.
36. Chettri N, Sharma E. Non-timber forest produce: utilization, distribution, and status in the Khangchendzonga Biosphere Reserve, Sikkim, India. In: Arrawatia ML, Tambe S, editors. Biodiversity of Sikkim: exploring and conserving a global hotspot. Department of Information and Public Relations, Government of Sikkim; 2011. p. 165-83.
37. Chettri N, Sharma E, Lama S. Non-timber forest produces utilization, distribution, and status in a trekking corridor of Sikkim, India. Lyonia. 2005; URL: http://www.lyonia.org/downloadPDF.php?pdfID=143.
38. Chhetri DR. Ethnomedicinal plants of the Khangchendzonga National Park, Sikkim, India. Ethnobot. 2005; URL: http://14.139.206.50:8080/jspui/handle/1/3649.
39. Chhetri DR. Herbal medicinal culture of the Nepalis of Darjeeling and Sikkim. In: Subba TB, Sinha AC, Nepal GS, Nepal DR, editors. Indian Nepalis: issues and perspectives. Gangtok: Concept Publishing; 2009. p. 311-2.
40. Chhetri DR, Parajuli P, Subba G. Anti-diabetic plants used by Sikkim and Darjeeling Himalayan tribes, India. J Ethnopharmacol. 2005;99:199-202. doi:10.1016/j.jep.2005.01.058.
41. Das K. Mushrooms of Sikkim: Barsey Rhododendron Sanctuary. Gangtok: Sikkim State Biodiversity Board; 2009.
42. Das K. Promising wild mushrooms from Sikkim Himalaya with ethnomycological significance and potentialities. In: Kumar S, editor. Ethnobotanical Studies in India. New Delhi: Deep Publications; 2014. p. 168-86.
43. Das T, Mishra S, Saha D, Agarwal S. Ethnobotanical survey of medicinal plants used by ethnic and rural people in Eastern Sikkim Himalayan Region. African J Basic Appl Sci. 2012;4(1):16-20. doi:10.5829/idosi.ajbas.2012.4.1.61133.

Additional File I

Bibliography of reviewed biocultural records from Sikkim

44. Dash SS. Useful plants of Kabi Sacred Grove, Sikkim. NELUMBO. 2007; doi:10.20324/nelumbo/v49/2007/73992.
45. Dash SS. Traditional herbal remedies used in Sikkim, India. NELUMBO. 2009; 5:123-56. doi:10.20324/nelumbo/v51/2009/58088.
46. Dash SS, Maiti A, Rai SK. Traditional uses of plants among the urban population of Gangtok, Sikkim. J Econ Taxon Bot. 2003;27(1):317-24.
47. Dhakal RD, Sharma G, Basnet K. Study of community dependence on Maenam Wildlife Sanctuary in the Sikkim Himalayas. J Hill Res. 2006;19(1): 24-33.
48. Government of India. Medicinal Plants, NTFP and Bamboos of Sikkim. Gangtok: ENVIS Centre on Conservation of Medicinal Plants; 2015. URL: http://www.sikkimforest.gov.in/medicine_main.htm.
49. Government of Sikkim. Medicinal plant species of conservation concern identified for Sikkim (SK). Gangtok: ENVIS Centre on Conservation of Medicinal Plants. 2014. URL: http://envis.frlht.org/documents/sikkim-medicinal-plants-2014.pdf.
50. Garg S, Patil U, Shrivastava RC. Wound healing potential of Viscum articulatum Burm., an ethmomedicinal plant of Sikkim on rat. Int J Res Phytochem Pharmacol. 2012;2(1):138-42. URL: http://www.indianjournals.com/ijor.aspx?target=ijor:rjpt&volume=6&issue=6&article=12.
51. Ghosh D, Mitra P, Ghosh T, Gupta S, Basu B, Mitra P. Isolation of emodin from the leaves of *Amaranthus spinosis* L. (Amaranthaceae). World J Pharma Res. 2014;3(6):1780-90.
52. Gulia BKS. Human ecology of Sikkim. New Delhi: Kalpaz Publications; 2005.
53. Gurung B. The medicinal plants of the Sikkim Himalaya. Chakung: Maples; 2008.
54. Gurung N, Pradhan S, Thapa G, Basistha B. Anti-hypoglycemic plants from western part of Sikkim Himalaya. Translation Med Biotech. 2014;2(5):13-23.
55. Hajra P, Chakravarty P. Medicinal plants of Sikkim and the Eastern Himalayas. Indian Forest. 1981;4(3):217-20.
56. Hooker JD. The Rhododendrons of the Sikkim-Himalaya. London: Bentham and Reeve; 1849.
57. Hooker JD. Himalayan journals: notes of a naturalist in Bengal, the Sikkim and Nepal Himalayas, the Khasia Mountains, etc. London: John Murray; 1855.
58. Hussain S, Hore D. Collection and conservation of major medicinal plants of Darjeeling and Sikkim Himalayas. Indian J Tradit Knowl. 2007;6(2):353-7. URL: http://www.niscair.res.in/sciencecommunication/researchjournals/rejour/ijtk/Fulltextsearch/2007/April%202007/IJTK-Vol%206%282%29-April2007-pp%20352-357.htm.
59. Idrisi M, Badola H, Singh R. Indigenous knowledge and medicinal use of plants by local communities in Rangit Valley, South Sikkim, India. NeBIO. 2010;1(2):34-45.
60. Jana S, Chauhan A. Studies on the Lepcha medico-botany of Dzongu in the Sikkim Himalaya. Himalayan Paryavaran. 1999;1:121-6.
61. Jana S, Chauhan A. Ethnobotanical studies on Lepchas of Dzongu, North Sikkim, India. Annals Forest. 2000;8(1):131-44.
62. Janmeda B, Lokendra S, Vats P. Folk claims on monocotyledonous plants in East Sikkim. Fitoterapia. 2006;70:155-60.
63. Jha V, Jha A. Traditional knowledge on disaster management: a preliminary study of Lepchas of Sikkim. Indian J Tradit Knowl. 2001;10(1):173-82. URL: http://nopr.niscair.res.in/handle/123456789/11078.
64. Jha A, Jha S, Suhag V. Traditional bamboo-based technology: a study of Lepchas of Sikkim. In: Proceedings of the Second International Congress of Chemistry and Environment. Indore. 2005. p. 250-251.
65. Jha A, Rao A, Jha S, Suhag V. A preliminary survey of plants used as food by Lepchas of Dzongu area in Sikkim, India. Crop Res. 2003;28(1):135-7.
66. Joshi V, Rawar MS, Sharma SK, Kumar K, Panda AK. Traditional knowledge of natural disaster mitigation and ethnomedicine practices in Himalaya with special reference to Sikkim. Indian J Tradit Knowl. 2011;10(1):198-206. URL: http://nopr.niscair.res.in/bitstream/.../11082/1/IJTK%2010(1)%20198-206.pdf.

67. Kapahi BK, Atal CK. Ethno-medico-botanical investigations in Sikkim. J Econ Taxon Bot. 1987;11(2):413-21.
68. Kholia BS. Traditional uses of pteridophytes in Sikkim Himalayas. In: Kumar S, editor. Ethnobotanical studies in India. New Delhi: Deep Publications; 2014. p. 291-302.
69. Krishna B, Das BK. Fibre-yielding plants of Sikkim. J Econ Taxon Bot. 1983;4:129-32.
70. Krishna B, Mitra B, Raju DCS. Yams (Dioscorea L.) in Sikkim. J Hill Res. 1988;1(2):67-72.
71. Krishna B, Singh S. Ethnobotanical observations in Sikkim. J Econ Taxon Bot. 1987;9(1):1-7.
72. Kumar A, Avasthe R, Shukla G, Pradhan Y. Ethnobotanical edible plant biodiversity of Lepcha tribes. Indian Forest. 2012;138(9):798-803.
73. Kumar R, Deo C. Biochemical evaluation of different Passion Fruit (Passiflora edulis) cultivars raised in Sikkim. Indian J Ag Sci. 2005;74(9):555-6.
74. Kumar S, Raju DCS. Large Cardamom and its wild relatives in Sikkim Himalayas. J Hill Res. 1998;2(2):375-9.
75. Kumar S, Singh P, Singh V. 1994. Ethnobotanical aspects of some arboreal and arborescent taxa of Sikkim. In: Gupta B, editor. Higher plants of the Indian sub-continent. New Delhi: BSMPS; 1994. p. 164-6.
76. Lachungpa U. Indigenous lifestyles and biodiversity conservation issues in North Sikkim. Ind J Tradit Knowl. 2009; URL: http://www.nopr.niscair.res.in/bitstream/123456789/.../IJTK 8(1) 51-55.pdf.
77. Lepcha L, Basistha B, Subba K, Rajdeep G, Sharma N. A reckon on the conservation and sustainability of *Abroma augusta* L. of Sikkim Himalaya. J Med Sci Res. 2012;3(2):25-9.
78. Lepcha S, Das A. Ethno-medico-botanical exploration along the international borders to Tibet Autonomous Region of China and the kingdom of Bhutan with special reference to the Pangolakha Wildlife Sanctuary, East Sikkim. In: Ghosh C, Das AP, editors. Recent studies in biodiversity and traditional knowledge in India. Malda: Gour College Press; 2011. p. 257-70.
79. Lepcha S, Gurung R, Arrawatia M. Traditional Lepcha craft Sumok-thyaktyk (Lepcha Hat) and its conservation in Dzongu Tribal Reserved Area (DTRA), Sikkim, India. Indian J Tradit Knowl. 2012;11(3):537-41. URL: http://www.nopr.niscair.res.in/bitstream/.../14398/1/IJTK%2011(3)%20537-541.pdf.
80. Lepcha L, Roy S, Basistha B, Sharma N, Subba K, Gurung R. Medicinal value and microbial VAM incidence analysis of Bischofia javanica Blume in Sikkim Himalaya, India. Photon. 2013;120:650-5.
81. Lepcha L, Roy S, Sarkar A, Basistha B, Arrawatia M. Documentation of medicinally important plants from the landslide prone areas of East Sikkim, India: a survey report. J Phytol. 2011;3(7):1−7. URL: http://scienceflora.org/journals/index.php/jp/article/view/2313/2291.
82. Lepcha S, Tamang P. Folk medicinal plants: threats and conservation approaches in Sikkim Himalaya. In: Sikkim biodiversity: significance and sustainability. Gangtok: Sikkim State Council of Science and Technology; 2012. p. 199-206.
83. Lobsang K, Jong N. Findings of the Chagpori Tibetan Medical Institute's herbal exploration excursion in West Sikkim. Darjeeling: Chagpori Tibetan Medical Institute; 1994.
84. Maity D, Chauhan A, Maiti G. Ethnobotanical notes on some unexploited medicinal plants used by Lepchas and Nepalese communities in North Sikkim. J Econ Taxon Bot; 2003:27(2):325-32.
85. Maity D, Jana SK, Mondal MS. Common Foxglove: a promising medicinal plant from Sikkim. In: Research Papers on Bioresources of Sikkim. Vol. 1. Gangtok: Government of Sikkim; 2000. p. 238-9.
86. Maity D, Pradhan N, Chauhan A. Folk uses of some medicinal plants from North Sikkim. Indian J Tradit Knowl. 2004;3(1):66-71. URL: http://www.nopr.niscair.res.in/bitstream/123456789/.../IJTK%203(1)%2066-71.pdf.
87. Manjerkar N. A study of local health traditions of Sikkim. Bangalore: Foundation for Revitalization of Local Health Traditions; 2000.
88. Mitra B. Srew-pine (*Pandanus nepalensis* St. John) in Sikkim Himalaya. J Hill Res. 1999;2(1):66-7.
89. Mudaiya RK, Tiwari RN, Majumdar R. Threatened and rare medicinal plants of Sikkim. Bull Medico-ethnobot Res. 1987; 15(1-2):24-6.

Additional File I

Bibliography of reviewed biocultural records from Sikkim

90. Mukhia B, Mukhopadhyay M. An ethnobotanical study on Limboos of West Sikkim. In: International Seminar on 'Multidisciplinary Approaches in Angiosperm Systematics'. Kalyani: University of Kalyani; 2012. p. 716-24.
91. Nath BG, Pathak PK, Mohanty AK, Tripathi AK. Management practices and performance of dairy cows in tribal areas of Sikkim. Asian Ag Hist. 2013;17(3):241-50.
92. Pal S, Palit D. Traditional knowledge and bio-resource utilization among Lepcha in North Sikkim. NeBIO. 2011;2(1):13-7.
93. Panda AK. Some folklore claims from the state of Sikkim. In: Biodiversity News Letter. India; 2007. p. 4-6.
94. Panda AK. Medicinal plants of Sikkim in Ayurvedic practice. Gangtok: Regional Research Institute; 2008.
95. Panda AK. Medicinal plants use and primary health care in Sikkim. Int J Ayurveda Herb Med. 2013;2(2):253-9.
96. Panda AK, Mandal D. The folklore medicinal orchids of Sikkim. Ancient Sci Life 2013;33(2):92-6. doi:10.4103/0257-7941.139043.
97. Panda AK, Mishra S. Some belief, practices, and prospects of folk healers of Sikkim. Indian J Tradit Knowl. 2012;11(2):369-73.
98. Panda AK, Mishra S. 2012. Plant biodiversity of Sikkim & Ayurvedic health care system. In: Sikkim biodiversity: significance and sustainability. Gangtok: Sikkim State Council of Science and Technology; 2012. p. 33-7.
99. Panda AK, Mishra S. Health traditions of Sikkim Himalaya. J Ayurveda Integr Med. 2010;1(3):183-9. doi:10.4103/0975-9476.72617.
100. Panda AK, Mishra S. Tracing the historical perspective of *Cordyceps sinensis*: an aphrodisiac of Sikkim Himalaya. J Ayurveda Integr Med. 2010;1:190-4.
101. Panda AK, Swain K. Traditional uses and medicinal potential of *Cordyceps sinensis* of Sikkim. J Ayurveda Integr Med. 2011;2: 9-13. URL: http://www.jaim.in/temp/JAyurvedaIntegrMed219-5648516_013408.pdf.
102. Panda S. Notes on ethnobotany of Ericaceae from the Eastern Himalayas and North-Eastern India. NELUMBO. 2008;50:105-10.
103. Pandey VN. Medico-ethno-botanical exploration in Sikkim. New Delhi: Central Council for Research in Ayurveda and Siddha; 1991.
104. Pradhan BK, Badola HK. Ethnomedicinal plant use by Lepcha tribe of Dzongu Valley, bordering Khangchendzonga Biosphere Reserve, in North Sikkim, India. J Ethnobiol Ethnomed. 2008; doi:10.1186/1746-4269-4-22.
105. Pradhan BK, Badola HK. Local knowledge on the use of Swertia chirayta as traditional medicine: conservation challenges in Sikkim Himalaya, India. Ethnobot Res Appl. 2015;14:345-55. doi:10.17348/era.14.0.345-355.
106. Pradhan BK. Caterpillar Mushroom, *Ophiocordyceps sinensis* (Ascomycetes): a potential bioresource for commercialization in Sikkim Himalaya, India. Int J Med Mushrooms. 2016;18(4):337-46. doi:10.1615/IntJMedMushrooms.v18.i4.70.
107. Pradhan H. Gorkha and other ethnic herbal medicines. New Delhi: Universal Publishers; 1991.
108. Pradhan KC. The Rhododendrons of Sikkim. Kolkata: Sikkim Adventure Botanical Tours and Treks; 2008.
109. Pradhan KC, Lachungpa ST. Sikkim-Himalayan Rhododendrons. Kalimpong: Primulaceae Books; 1990.
110. Pradhan S, Basistha B, Basnett R, Banerjee A. Chromatographic techniques used for investigation of basic bioactive constituents of highly potent species—*Hippophae salicifolia* from hidden forests of Sikkim. In: Sikkim biodiversity: significance and sustainability. Gangtok: Sikkim State Council of Science and Technology; 2012. p. 80-91.
111. Pradhan S, Tamang J. Ethnobiology of wild leafy vegetable of Sikkim. Indian J Tradit Knowl. 2015;12(2):290-7. URL: http://14.139.47.15/bitstream/123456789/32086/1/IJTK14(2) 290-297.pdf.
112. Puri H, Pandey G. Glimpses into the crude drugs of Sikkim. Bull Medico-ethnobot Res. 1980;1(1):55-71.
113. Purohit CS, Agarwala DK, Das K. Ethno-botanical plants of Sikkim and their status: a review. In: Kumar S, editor. Ethnobotanical Studies in India. New Delhi: Deep Publications; 2014. p. 256-77.

Additional File I

Bibliography of reviewed biocultural records from Sikkim

114. Rai LK, Sharma E. Medicinal plants of the Sikkim Himalaya: status, uses and potential. Dehra Dun: Bishen Singh Mahendra Pal Singh; 1994.
115. Rai LK, Prasad P, Sharma E. Conservation threats to some important medicinal plants of the Sikkim Himalaya. Biol Conserv; 2010. doi:10.1016/S0006-3207(99)00116-0.
116. Rai, PC. The practice of traditional healing and the healers of Ribdi-Bhareng. Gangtok: Village Health Association of Sikkim; 2010.
117. Rai P, Sarkar A, Das A. Ethnomedicinal studies in some fringe areas of Sikkim and Darjeeling Himalaya. J Hill Res. 1998;11:12-21.
118. Rai S, Bhujel R. Ethnic uses of some monocotyledonous plants in the Darjeeling Himalayan region. In: Das A, editor. Perspectives of plant biodiversity. Dehra Dun: Bishen Singh Mahendra Pal Singh; 2002. p. 635-44.
119. Rai B, Khawas DB, Chettri R. Certain plants in the folklore and folklife of Darjeeling and Sikkim hills, India used for treatment of human ailments. J Econ Taxon Bot. 1992;10:193-8.
120. Rai T, Rai LK. Trees of the Sikkim Himalaya. New Delhi: Indus Publishing Company; 1994.
121. Rai YK, Rai LK. *Citrus reticulata* in the Mamlay watershed, agroforestry practice, and market. J Hill Res. 1994;7(2):157-60.
122. Rai YK, Singh KK, Rai LK. Diversity of edible wild plants in the Sikkim Himalaya. In: Sikkim biodiversity: significance and sustainability. Gangtok: Sikkim State Council of Science and Technology; 2012. p. 3-19.
123. Rajendran, SM. West Sikkim: a treasure house of medicinal plants and their utility by local tribals. J Econ Taxon Bot. 2003;27:311-6.
124. Raju D, Mandal N. Native *Kutki* (*Picorhizza scropularifolia*) of Sikkim. J Hill Res. 1990;3:123-6.
125. Raju D, Krishna B. Less known edible plants of Sikkim. In: Porkayastha R, editor. Economic Plants and Microbes. Kolkata: Today and Tomorrow's Printers and Publishers; 1990.
126. Raju D, Sanjapp M, Singh S. *Ma Niao Pao* in Sikkim. J Hill Res. 1990;3:131-4.
127. Royburman JJ. Tribal medicine: traditional practices and change in Sikkim. New Delhi: Mittal Publications; 2003.
128. Sahoo A, Ansari A. Less known ethnic uses of plants of South Sikkim. NELUMBO. 2009;51: 219-22.
129. Saklani A, Upreti D. Folk uses of some lichens in Sikkim. J Ethnopharmacol. 1992; doi:10.1016/0378-8741(92)90038-S.
130. Sharma TP, Borthakur SK. 2008. Traditional handloom and handicrafts of Sikkim. Indian J Tradit Knowl. 2008;9(2):375-7. URL: http://nopr.niscair.res.in/bitstream/123456789/8188/1/IJTK%209(2)%20375 377.pdf?utm_source=The_Journal_Database&trk=right_banner&id=1416745567&ref=a a74080be9b3f3036a13c3678593fb0.
131. Sharma E, Rai L, Lachungpa S, Awasthi R. Status of medicinal plants and their cultivation potential in Sikkim. In: Sundriyal R, Sharama E, editors. Cultivation of medicinal plants and orchids in Sikkim Himalaya. Dehra Dun: G. B. Pant Institute of Himalayan Environment and Development; 1995. p. 43-51.
132. Sharma T. Diversity of non-timber forest produce (NTFP) in Sikkim Himalaya: an overview. In: Sikkim biodiversity: significance and sustainability. Gangtok: Sikkim State Council of Science and Technology. p. 92-5.
133. Sharma T, Sharma S. Medicinal plants of Sikkim. Gangtok: Beracah Printing and Stationary; 2010.
134. Sharma BD, Srivastava RC. Medicinal plants of Sikkim. Kolkata: Botanical Survey of India, 1993.
135. Sherpa MT, Mathur A, Das S. Medicinal plants and traditional medicine system of Sikkim: a review. World J Pharma Res. 2015;4(2):161-81.
136. Shrestha B, Basnett H, Prosanta P. Herbal remedies practiced by traditional practitioners of Nepali tribe in Sikkim. Uni J Pharma Sci Res. 2015; http://ujpsr.com/sites/default/files/articles/UJPSRMNSC-1 %281%29.pdf.
137. Singh HB, Sharma BR, Pradhan B. Ethnobotanical observation on the preparation of *rokshi* (a local drink) in Sikkim, India. J Econ Taxon Bot. 1999;23(2):580-2.

138. Singh D. Use of medicinal plants of Sikkim in Ayurvedic medicine. In: Sundriyal R, Sharma E, editors. Cultivation of medicinal plants and orchids in Sikkim Himalaya. Dehra Dun: Bishen Singh Mahendra Singh; 1995. p. 65-8.
139. Singh HB, Jain A. Ethnobotanical observation on the preparation of Millet beer in Sikkim state, India. J Econ Taxon Bot. 1999;23(2): 577-9.
140. Singh H, Prasad P, Rai L. Folk medicinal plants in the Sikkim Himalayas of India. Asian Folklore Studies. 2002;61:295-310. doi:10.2307/1178975.
141. Singh P. Medicinal plants of Sikkim and the Eastern Himalayas: a practical analysis about clinical characters of North East India's medicinal plants. New Delhi: Spectrum; 2007.
142. Singh V. Lesser known wild edibles of Sikkim Himalaya. J Econ Taxon Bot. 1995;19(2):385-90.
143. Singh VP. Some medicinal ferns of Sikkim Himalayas. JREIM. 1973;8(3):71-3.
144. Sinha G, Chauhan A. Ethnobotanical studies on Lepchas of Sikkim Himalaya. Himalayan Paryavaran. 1997;5(1):60-4.
145. Srivastava RC. High altitude medicinal plants of Sikkim Himalaya. JREIM. 1993;12(1):5-14.
146. Srivastava RC. Wild edible plants of Sikkim Himalaya. NELUMBO. 1994;36:95-126.
147. Srivastava RC. A note on the arrow-poison used by Lepchas of Dzongu (Sikkim) and its antidote. National Academy of Science Letters. 1990;13(11):399-400.
148. Srivastava TN, Kapaki BK. Resource survey of plants of potential economic value of Sikkim Himalayas. Bulletin of Medico-ethnobotanical Research. 1990;12(1-2):1-11.
149. Srivastava TN, Kapaki BK, Atal CK. Ethnomedico-botanical investigations in Sikkim. Journal of Economic and Taxonomic Botany. 1987;11(2):413-21.
150. Subba JR. Biodiversity of the Sikkim Himalaya. New Delhi: Ambica Printers; 2002.
151. Subba JR. Indigenous knowledge on bio-resources management for livelihood of the people of Sikkim. Indian J Tradit Knowl. 2009;8(1):56-64.
152. Subba S, Badola H. Ethnobotanical knowledge, populations, and *ex-situ* conservation trials in *Juglans regia* L. (Juglandaceae) in Sikkim. Pleione. 2011;5(2):304-11.
153. Sundriyal M, Rai LK. Wild edible plants of the Sikkim Himalaya. J Hill Res; 1996;9(2):267-78.
154. Sundriyal M, Sundriyal R. Underutilized edible plants of the Sikkim Himalaya: need for domestication. Cur Sci. 2003; http://www.iisc.ernet.in/currsci/sep252003/731.pdf.
155. Sundriyal M, Sundriyal R. Wild edible plants of the Sikkim Himalaya: marketing, value addition and implications for management. Econ Bot. 2004; doi:10.1163/0013-0001(2004)058.
156. Sundriyal M, Sundriyal R. Seedling growth and survival of selected wild edible fruit species of the Sikkim Himalaya, India. Acta Oecologica. 2005; doi:10.1016/j.actao.2005.02.003.
157. Sundriyal M, Sundriyal R, Sharma E. Dietary use of wild plant resources in the Sikkim Himalaya, India. Econ Bot. 2004;58(4):626-38.
158. Sundriyal M, Sundriyal R, Sharma E, Purohit A. Wild edibles and other useful plants from the Sikkim Himalaya, India. Oecol Mont. 1998;7(1-2):43-54.
159. Suresh C, Bhutia K, Shukla G, Pradhan K, Chakravarty S. 2014. Wild edible tree fruits of Sikkim Himalayas. J Tree Sci 33(1):43-7.
160. Talukdar D, Talukdar T. 2012. Traditional legumes in Sikkim Himalayas: food preparation, uses, and ethno-medicinal perspectives. Int J Cur Res 4(4):64-73.
161. Tamang B, Tamang J. Traditional knowledge of bio-preservation of perishable vegetable and bamboo shoots in Northeast India as food resources. Ind J of Tradit Knowl. 2009; http://www.niscair.res.in/sciencecommunication/researchjournals/rejour/ijtk/Fulltextsearh/2009/January 2009/IJTK-Vol 8(1)- January 2009- pp 89-95.htm.
162. Tamang JP, Thapa S, Tamang N, Rai B. Indigenous fermented food beverages of Darjeeling hills and Sikkim: process and product characterization. J Hill Res. 1996;9(2):401-11.
163. Tamang K, Yonzone G. Dictionary of flowering plants of Darjeeling-Sikkim Himalaya. Darjeeling: Goodwill Home; 2004.

Additional File I

Bibliography of reviewed biocultural records from Sikkim

164. Tamang P. Ichthyo-faunal diversities of Sikkim: Taxonomic list, fisheries trend, and conservation exigencies. In: Sikkim biodiversity: significance and sustainability. Gangtok: Sikkim State Council of Science and Technology; 2012. p. 170-9.
165. Tambe S, Rawat G. Traditional livelihood based on sheep grazing in Khangchendzonga National Park of Sikkim. In: Sikkim biodiversity: significance and sustainability. Gangtok: Sikkim State Council of Science and Technology; 2012. p. 158-66.
166. Tamsang KP. 2004. Glossary of Lepcha medicinal plants. Kalimpong: Mani Printing Press; 2004.
167. Thapa K, Chettri R. Ethnobotanical survey of Darjeeling-Sikkim hills. Bulletin of Cell Research-India. 1993;1(2):1-7.
168. Tripathi KP, Singh B, Jain RK. Characterization of medicinal plants used by different tribes in the Sikkim Himalayas. J NTFP. 2009;16(2):111-8.
169. Tsarong T. Tibetan medicinal plants: an agenda for cultivation. In: Sundriyal R, Sharma E, editors. Cultivation of medicinal plants and orchids in Sikkim Himalaya. Dehra Dun: G. B. Pant Institute of Himalayan Environment and Development; 1995. p. 75-9.
170. Uniyal MR. Traditional useful medicinal plants of Sikkim Himalaya. Professional Seminar on Research on Ayurveda and Siddha; 1995: 76.
171. Upadhyaya RC, Patiram RS. Decline status of Mandarin Orange (*Citrus reticulata* Blanco) in Sikkim. J Hill Res. 1994;7(2):83-9
172. Upadhyaya RS, Patiram. Nutrient status of Mandarin Orange (*Citrus reticulata* Blanco) in Sikkim. Pages 83−87 in Research Papers on Bioresources of Sikkim. Volume 1. Gangtok: Government of Sikkim; 2000. p. 83-7.
173. Ved DK, Kinhal GA, Haridasan K, Ravikumar K, Ghate U, Sankar RV, *et al.* Conservation assessment and management prioritization for the medicinal plants of Arunachal Pradesh, Assam, Meghalaya and Sikkim. Bangalore: Lotus Enterprises, Bangalore; 2003.
174. Yonzan H, Tamang JP. Consumption pattern of traditional fermented foods in the Sikkim Himalaya. J Hill Res. 1998;11(1): 112-5.
175. Yonzone G, Bharati P, Yonzan B, Bhujel R. Ethnomedicinal plants of Darjeeling–Sikkim Himalayas. J Int Hort Soc. 1987;1:193-202.
